# Current perceptions and working time models of female urologists in Germany: results of a large-scale survey

**DOI:** 10.1007/s00345-023-04604-8

**Published:** 2023-09-13

**Authors:** Sarah Weinberger, Maria-Noemi Welte, Sophie Knipper, Carolin Siech, Eva Maria Greiser, Laura Wiemer, Karina Müller, Laura Bellut, Annika Herlemann, Sandra Schoenburg, Margarete Teresa Walach

**Affiliations:** 1 Department of Urology, Charité, University Medicine Berlin, Berlin, Germany; 2 Department of Urology, University Hospital Frankfurt, Frankfurt, Germany; 3 Martini-Klinik Prostate Cancer Center, University Hospital Hamburg-Eppendorf, Hamburg, Germany; 4 Department of Urology, Mathias-Spital, Hospital Rheine, Rheine, Germany; 5 Pro Uro Berlin, Berlin, Germany; 6 Department of Urology, Hospital Bremen Mitte, Bremen, Germany; 7 Department of Urology, University Hospital Erlangen, Erlangen, Germany; 8 Department of Urology, Campus Großhadern, LMU Munich, Munich, Germany; 9 Department of Urology and Kidney Transplantation, Martin Luther University, Halle, Germany; 10 Department of Urology and Urosurgery, University Medical Centre Mannheim (UMM), University of Heidelberg, Theodor-Kutzer-Ufer 1-3, 68167 Mannheim, Germany

**Keywords:** Female urologist, Working time models, Part-time work, Survey

## Abstract

**Purpose:**

Female urologists are distinctly underrepresented in leading positions. The reasons behind this inequity remain unclear, with some suggesting factors such as family responsibilities, part-time work and insufficient mentorship. This study aimed to explore and characterize the working conditions of female urologists in Germany, with a focus on factors influencing the working time model.

**Methods:**

A questionnaire was developed and distributed to 1343 female members of the German Society of Urology between February and March 2022. The survey consisted of 43 questions covering the categories demographics, occupation situation, satisfaction at work, family situation, career aspects and research activity.

**Results:**

Of the 487 female German urologists who participated in the survey, 167 (34.3%) worked part-time. Doctors in training were significantly less likely to work part-time than colleagues who had completed their specialist training (*p* < 0.001). Only 10% of female doctors in training reported working part-time. Similarly, having children (*p* < 0.001) and engaging in scientific activities (*p* = 0.03) were independent factors influencing part-time work, with children increasing the likelihood of working part-time as expected, while scientifically active female urologists were more likely to work full-time.

**Conclusion:**

This study provides the largest survey on the situation of female urologists in German-speaking countries to date. Part-time work during specialist training is rare, while more than 50% of female urologists with children work part-time. With the projected decline in the number of practicing physicians and the increasing demand for medical attention, it is crucial to find ways to retain and support healthcare professionals, particularly female urologists.

## Introduction

The proportion of women in medicine and surgery has increased steadily over the last few decades. More than half of all medical students across many countries are female [1–3]. However, there is a significant contrast when it comes to the number of female surgeons, including those in the subspecialty of urology. In 2020, female urologists accounted for only 10.3% of all urologists in the USA [4]. In Germany, the number of female urologists is still relatively small compared to the number of male colleagues, but the gender ratio is becoming more and more balanced. The proportion of practicing female urologists increased from 11.3% in 2008 to 20.6% in 2021 [5]. Interestingly, the proportion of female residents in urology in Germany was 55% in 2021 [5], but this percentage drops to 38.3% among female specialists in urology. In academic leadership positions, female urologists are distinctly underrepresented compared to male urologists [5]. There is limited evidence on the reasons for this inequality. The most commonly proposed factors in the field of surgery include family responsibilities [6, 7], maternity leave, part-time work, but also a lack of interest [8] and an insufficient mentorship [9]. Individual working-time arrangements are increasing globally, and the traditional vertical career path is becoming more flexible. However, in the medical sector, part-time work is gendered to a large extent, with most part-time workers being female employees [10, 11]. It remains unclear whether the decision to reduce working hours is made voluntarily or if women are forced to reduce working hours due to working conditions and private circumstances.

In this study, we aimed to explore and characterize the working conditions, including surgical activity, professional status, and goals of female urologists in Germany, with a focus on influencing factors on the chosen working model. Our central question was: where does urology in Germany stand up to date in terms of female representation, current perceptions, and challenges in gender disparity? To our best knowledge, this is the largest survey on this topic conducted not only in the German-speaking countries but also to date worldwide.

## Materials and methods

Between November 2021 and February 2022, a working-group of 11 female urologists created a questionnaire to survey the current professional situation of female urologists in German speaking countries. The questionnaire was sent to 1343 female members of the German society of Urology (Deutsche Gesellschaft für Urologie, DGU). It was distributed via the DGU email list on February 28, 2022, and could be answered anonymously until March 30, 2022.

### Questionnaire and endpoint

The questionnaire consisted of 43 questions, including multiple-choice questions, yes and/or no style questions, ranking style questions and open text boxes. The survey consisted of six main categories: demographic information, occupation situation, satisfaction at work, family situation, career aspects and research activity. The primary aim of this study was a descriptive presentation of the situation of female urologists in Germany. Additionally, we performed analyses regarding correlations between the working model and influencing factors on part-time work.

### Statistical analyses

Descriptive statistics included frequencies and proportions for categorical variables. Means, medians, and ranges were reported for continuously coded variables. The statistical significance of differences in medians and proportions was assessed with the Wilcoxon rank sum test; Pearson’s Chi-squared test; Fisher’s exact test. Normal distribution was assessed with graphical methods via histograms. A *p* value of ≤ 0.05 was considered statistically significant. Uni- and multivariable logistic regression analyses (backward selection) were performed to evaluate influencing factors on part-time work. All analyses were performed using the software environment R version 3.4.3 (R project, R Foundation for Statistical Computing, Vienna, Austria).

## Results

Of 1343 recipients in Germany, Austria and Switzerland, 521 female urologists answered the questionnaire, which corresponds to a response rate of 38.8%. The average completion time for the survey resulted in 8:57 min. For the present study, we included the answers of 487 participants working in Germany. Table 1 shows the answers of all 487 participants regarding private life, occupation, satisfaction at work, family situation, career aspects and research activity. Working full-time was reported by 303 of 487 (62%) survey participants, whereas 167 (34%) reported to work part-time. Participants working part-time were statistically significantly older, more likely in a relationship and had more likely children. Concerning career aspects, residents were more likely to work full-time (128/163; 78.5%). Part-time work was most widespread among attending physicians (78/137; 56.9%). 29.5% of senior attending physicians and 17.2% of residents reported working part-time, while none of five chairs of department were working part-time. Female urologists working part-time stated significantly less overtime hours per week and less night shifts per month than their colleagues working full-time. But they were also more likely not performing surgery. Furthermore, part-time workers were more likely to feel unequally treated at work. Concerning research activity, part-time workers had less often a doctoral degree or postdoctoral qualification and were less likely to pursue research activities.

**Table 1:** Characteristics of all participants

	All participants	Full-time working	Part-time working	*p* value
Number of participants, *n*	487	303 ^+^	167 ^+^	
Age in years, median (IQR)	37 (33–44) *	35 (31–42)	41 (36–47)	< 0.001
Age categories, *n* (%)				< 0.001
< 30 years	68 (14)	63 (21)	4 (2.4)	
31–40 years	238 (49)	154 (51)	74 (44)	
41–50 years	100 (21)	38 (13)	60 (36)	
51–60 years	66 (14)	40 (13)	24 (14)	
> 60 years	13 (2.7)	6 (2)	5 (3)	
Data n/a	2 (0.4)	2 (0.7)	0 (0.0)	
Stable relationship, *n* (%)				0.001
Yes	406 (84)	239 (79)	151 (90)	
No	80 (16)	64 (21)	16 (9.6)	
Data n/a	1			
Children, *n* (%)				< 0.001
Yes	260 (54)	94 (31)	151 (90)	
No	224 (46)	207 (69)	16 (9.6)	
Data n/a	3	2		
Career stage, *n* (%)				< 0.001
Resident	163 (33)	128 (42)	28 (17)	
Attending physician	137 (28)	53 (17)	78 (47)	
Senior attending physician	95 (20)	64 (21)	28 (17)	
Chair of department	5 (1)	5 (1.7)	0 (0.0)	
Private practice/office	81 (17)	49 (16)	32 (19)	
Data n/a	6 (1.2)	4 (1.3)	1 (0.6)	
Hospital’s level of care, *n* (%)				< 0.001
Primary/secondary care, non-academic	146 (30)	85 (28)	56 (34)	
Tertiary care, non-academic	115 (24)	73 (24)	36 (22)	
University hospital	106 (22)	84 (28)	19 (11)	
Data n/a	120 (25)	61 (20)	56 (34)	
Sector of workplace, *n* (%)				< 0.001
Outpatient services	169 (35)	92 (30)	73 (44)	
Inpatient care	347 (71)	233 (77)	105 (63)	
Private industry	7 (1.4)	4 (1.3)	2 (1.2)	
Public/state authority	8 (1.6)	3 (1)	5 (3)	
Others	24	10 (3)	7 (4.2)	
Satisfaction with workplace, *n* (%)				0.4
Completely satisfied	101 (21)	67 (22)	31 (19)	
Rather satisfied	155 (32)	103 (34)	49 (29)	
Partly satisfied	142 (29)	85 (28)	50 (30)	
Rather unsatisfied	60 (12)	33 (11)	26 (16)	
Completely unsatisfied	27 (5.5)	14 (4.6)	11 (6.6)	
Data n/a	2 (0.4)	1 (0.3)	0 (0.0)	
Satisfaction with specialization in urology, *n* (%)				0.4
Completely satisfied	357 (73)	231 (76)	117 (70)	
Rather satisfied	85 (17)	46 (15)	34 (20)	
Partly satisfied	36 (7.4)	20 (6.6)	14 (8.4)	
Rather unsatisfied	4 (0.8)	2 (0.7)	2 (1.2)	
Completely unsatisfied	3 (0.6)	3 (1.0)	0 (0.0)	
Data n/a	2 (0.4)	1 (0.3)	0 (0.0)	
Satisfaction with working time model, *n* (%)				0.10
Completely satisfied	136 (28)	75 (25)	57 (34)	
Rather satisfied	170 (35)	113 (37)	55 (33)	
Partly satisfied	118 (24)	75 (25)	38 (23)	
Rather unsatisfied	45 (9.2)	28 (9.2)	16 (9.6)	
Completely unsatisfied	5 (1)	4 (1.3)	1 (0.6)	
Data n/a	13 (2.7)	8 (2.6)	0 (0.0)	
Overtime hours/week, median (IQR)	5 (3–10)	8 (5–10)	4 (2–5)	< 0.001
Data n/a	101	72	19	
Night shifts and 24h shifts/month, median (IQR) Data n/a	4 (0–6) 100	4 (3–6) 59	2 (0–4) 30	< 0.001
Experience of unequal treatment at workplace, *n* (%)				0.006
Yes	352 (73)	209 (70)	135 (81)	
No	128 (27)	91 (30)	31 (19)	
Data n/a	7	3	1	
Surgical activity, *n* (%)				< 0.001
Yes	174 (36)	117 (39)	52 (31)	
No	110 (23)	44 (15)	60 (36)	
Data n/a	203 (42)	142 (47)	55 (33)	
Complex surgeries^a^, *n* (%)				0.5
Yes	75 (19)	54 (20)	21 (17)	
No	322 (81)	214 (80)	101 (83)	
Data n/a	90	35	45	
Doctoral degree, *n* (%)				< 0.001
Yes	334 (69)	210 (69)	113 (68)	
No	91 (19%)	43 (14)	44 (26)	
No, but in progress	61 (13)	50 (17)	10 (6)	
Data n/a	1 (0.2)	0 (0.0)	0 (0.0)	
Postdoctoral qualification, n (%)				< 0.001
Yes	11 (2.3)	21 (6.9)	3 (1.8)	
No, but in progress	50 (10)	43 (14)	7 (4.2)	
No, not planned	401 (82)	232 (77)	154 (92)	
Data n/a	11 (2.3)	7 (2.3)	3 (1.8)	
Research activity, *n* (%)				< 0.001
Yes	117 (24)	97 (32)	18 (11)	
No, but planned in the future	48 (9.9)	33 (11)	14 (8.4)	
No, not planned	318 (65)	172 (57)	133 (80)	
Data n/a	4 (0.8)	1 (0.3)	2 (1.2)	
Career goals reached, *n* (%)				0.7
Yes	147 (30)	91 (30)	50 (30)	
No	163 (33)	96 (32)	61 (37)	
Not yet	170 (35)	111 (37)	54 (32)	
Data n/a	7 (1.4)	5 (1.7)	2 (1.2)	

^+^Data of 5 participants missing and 12 participants unemployed, *data of two participants missing; n number, IQR interquartile range, n/a not available

^a^Complex surgery—defined as: major uro-oncological surgeries, complex reconstructive interventions, transplantations

Univariable logistic regression analysis identified numerous factors influencing the probability of part-time work (Table 2). Higher age (OR 1.1; 95% CI 1.0–1.1, *p* < 0.001), children (OR 20.8; 95% CI 12.1–38.0; *p* < 0.001), and a relationship (OR 2.6; 95% CI 1.5–2.7; *p* = 0.002) significantly increased the likelihood of part-time work, while residents were significantly less likely to work part-time than colleagues who had successfully completed residency training. The absence of a postdoctoral qualification (OR 4.1; 95% CI 1.4–17.5; *p* = 0.02), research activity (OR 3.9; 95% CI 2.3–6.9; *p* < 0.001) and surgical activity (OR 3.1; 95% CI 1.9–5.1; *p* < 0.001) showed a positive correlation with the probability of part-time work. And colleagues with experience of unequal treatment at workplace were also more likely working part-time (OR 0.5; 95% CI 0.3–0.8; *p* = 0.007).

**Table 2:** Uni- and multivariable analyses of factors influencing part-time work

	Univariable analysis			Multivariable analysis		
	OR	95% CI	*p* value	OR	95% CI	*p* value
Part-time work						
Age (continuous)	1.1	1.0–1.1	**< 0.001**	1.0	1.0–1.1	0.8
Children (yes vs. no)	20.8	12.1–38.0	**< 0.001**	17.7	8.1–42.5	**< 0.001**
Relationship (yes vs. no)	2.6	1.5–2.7	**0.002**	1.0	0.4–2.5	0.9
Career Resident	Ref					
Attending physician	6.7	4.0–11.7	**< 0.001**	4.2	1.5–12.1	**0.007**
Senior attending physician	2.0	1.1–3.7	**0.02**	1.3	0.4–4.3	0.6
Chair of department	0.0	n/a	1.0	0.0	n/a	0.9
Medical practice	3.0	1.6–5.5	**< 0.001**	0.7	0.3–2.5	0.5
Level of care Primary/secondary care hospital	Ref					
Maximum care hospital	0.7	0.4–2.3	0.28	1.0	0.4–2.7	0.9
Medical practice	1.4	0.8–2.3	0.19	0.8	0.3–2.2	0.6
University hospital	0.3	0.2–0.6	**< 0.001**	0.8	0.2–3.2	0.7
Doctoral degree (no vs. yes)	1.1	0.7–1.6	0.70	–	–	–
Research activity (no vs. yes)	3.9	2.3–6.9	**< 0.001**	3.7	1.2–12.3	**0.03**
Surgical activity (no vs. yes)	3.1	1.9–5.1	**< 0.001**	2.0	0.9–4.2	0.08

*OR* odds ratio, *CI* confidence interval, *n/a* not available

Multivariable logistic regression analysis (Table 2) identified having children (OR 17.7; CI 8.1–42.5; *p* < 0.001), being an attending physician (OR 4.2; CI 1.5–12.7; *p* = 0.007) and not following research projects (OR 3.7; CI 1.2–12.3; *p* = 0.03) as significant predictive factors for part-time work. Relationship status, level of care of the hospital and surgical activity of the female urologist did not reach independent predictor status for part-time work.

## Discussion

In the future, the number of people requiring medical attention is expected to increase while the number of practicing physicians is projected to decline. Aging German society poses a dual demographic challenge for one of the world's oldest communities.

The German Medical Association's physician data for 2021 and 2022 indicates that approximately 46% of doctors are over 50 years old and will likely need to be replaced within the next 10 to 15 years [12, 13]. In 2021, about 55% of urologists were over 50 years old [12]. Moreover, fewer physicians, both men and women, are willing to work full-time [14, 15].

According to statistics from the microcensus of the Federal Statistical Office, there has been a significant increase in the number of physicians working part-time in Germany over the last decade. In 2012, approximately 15.8% of physicians worked part-time, whereas in 2022, this figure rose to 30.9% [16].

Regarding the gender distribution, the data indicates a rise in the proportion of both female and male physicians working part-time in recent years. In 2012, 26.4% of female physicians worked part-time, while in 2022, that proportion increased to 43.4% [16]. Similarly, the proportion of male physicians working part-time has also risen from 4.6% in 2012 to 15.5% in 2022 [16]. Our study confirms this trend, with 34% of female participants working part-time. A 2022 study by Schmedding et al. found that the rate of female pediatric surgeons working part-time was even higher, with 62% of women of whom 68% had underage children [17].

Balancing work and childcare responsibilities can be a significant challenge, and part-time work may be the most feasible option for some parents. However, part-time work can also limit career opportunities and earning potential, which can have long-term consequences for financial stability.

A study conducted by the German Federal Institute for Population Research found that women with children are more likely to work part-time than women without children. In 2020, 65.5% of mothers with children under the age of 18 worked part-time, compared to 7.1% of fathers [18]. The study also showed that part-time work was associated with lower wages and fewer opportunities for career advancement, particularly for women [18].

In line with these results, Baptiste et al. showed that female surgeons are more likely to be responsible primarily for childcare and household duties than their male colleagues [19]. Our study confirms that children are an independent predictor for part-time work.

A Dutch study on career perspectives among different medical specialties showed that women and younger men prefer to have the option of working part-time [20]. This study observed that the most female physicians working part-time had a partner and/or children while full-time working female doctors were more likely to be single and childless [20]. In another survey, personal circumstances and preferences, such as the importance of family and leisure pursuits, were reasons for working part-time, and surgeons were the least likely to work part-time [21]. Part-time working female physicians might be perceived as less committed to their work and career [10].

Another German survey of male and female urologists revealed that not only women with children prefer part-time work. Of the participants, 27.0% of women and 11.5% of men reported currently working part-time. However, 32.3% of the men and 55.6% of the women had a desire to work part-time [22].The percentage of women who wanted to work part-time was already about 50% in the Graduate Report on Medicine almost 30 years ago, so there has been no real change [11]. Interestingly, the study indicates a significant shift in the attitudes of males towards part-time work. 30 years ago, only 19% of males were interested in working part-time, whereas in this survey, 32.3% expressed their desire for it [22]. This shift reflects a generational conflict, with younger generations seeking a better work-life balance rather than complete self-sacrifice at work. However, this change is often misunderstood by the older generation, who may interpret it as disinterest or reluctance to work. Men also find it difficult to communicate their desire for part-time work to their superiors due to fear of being misunderstood or negatively impacting their job prospects.

The State Chamber of Physicians survey provides further evidence of this problem, with the number of doctors who are unhappy with their jobs increasing in Germany. More than a third of doctors intend to reduce their working hours, citing a need for a better work-life balance, a decrease in workload, and, deduced from the above mentioned references, more time for childcare [23].

Our data reveal that female urologists who work part-time are more likely to drop out of important subspecialties such as surgery and research activities. This issue is supported by a study conducted by Park et al. in 2017, which found that women surgeons were less likely to hold advanced degrees, leadership positions, and research involvement compared to their male counterparts. The study also reported higher rates of part-time work among female surgeons, which was linked to lower research productivity and fewer leadership opportunities for both men and women.

Promoting women and part-time workers in these fields can help reduce the experience of unequal treatment in the workplace, particularly in urology where the prevalence of gender bias is higher among part-time workers. The 2018 AUA census highlighted that 39% of women urologists experienced gender bias in their practice, whereas only 1.2% of male respondents reported the same issue [24].

The task of employers should, therefore, be to reduce these differences between women and men and half- and full-time workers. Employers play an important role in supporting part-time urologists by offering flexible scheduling, pro-rated salaries, and opportunities for leadership and research. This could also increase the esteem in which employees are held. With increasing esteem, the migration of physicians to other fields or abroad could also be reduced [25]. In the year 2021, the number of physicians in Germany in other fields of activity, for example in the industry, grew (+ 9.0%) in comparison to the years before [12].

Our study reveals that the rate of part-time work increases with age among female urologists: 29.5% of senior attending physicians and 17.2% of residents reported working part-time. In Germany, residents have the option to work part-time. However, choosing part-time work may extend the duration of their specialization beyond the standard 5-year period. This could explain why attending physicians, who have completed their specialization, are less likely to work part-time compared to residents. As physicians progress in their careers and approach retirement age, they may prioritize a better work-life balance and family responsibilities. This generational difference highlights the changing attitudes towards work and life preferences, which may have implications for workforce planning and supporting healthcare professionals throughout their careers.

Our study is not devoid of limitations with the main limitation being that the survey was only sent to female DGU members. Thus, the currently still larger percentage of active physicians in the urologic field was not taken into account and comparisons between genders were not possible. Moreover, non-DGU members were not included in the survey. In addition, as the survey was designed as a cross-sectional study with only a single time point, developments over time were not assessed. The response rate of 38.8% in this survey is comparable to other physician specialist survey-based studies but it is lower than the average response rates of surveys conducted in the general population [26–28]. Generally, the higher the response rate the more reliable and robust the data. But if the participants are representative of the population, good validity might still be reached. Thus, further studies on this topic with higher response rates are necessary. Nevertheless, we believe our results are still robust because of the representative cohort.

## Conclusion

Especially under women, but also for men, an appropriate working time model is becoming more and more crucial. To counteract the shortage of skilled workers, part-time work should be better accepted and integrated into professional life. This means that working part-time should not automatically exclude one from surgical activities or research projects.

Furthermore, part-time work should not lead to unequal treatment or discrimination in terms of opportunities and resources. By embracing and accommodating part-time work, more individuals can pursue a career in their desired field without sacrificing their work-life balance or personal obligations.

## Data Availability

Most data generated or analyzed during this study are included in this article. Further enquiries can be directed to the corresponding author.
